# Decoding cultured meat manufacturing: a full process model to identify scale-up bottlenecks

**DOI:** 10.3389/fnut.2026.1844185

**Published:** 2026-06-29

**Authors:** Katharina Julia Brenner, Jan Harvey Lindermann, Tjaša Sušnik, Alejandra Riera Hipp, Sonja Berensmeier, Thomas Hamacher, Chantal Treinen, Marius Henkel

**Affiliations:** 1 Cellular Agriculture, TUM School of Life Sciences, Technical University of Munich, Freising, Germany; 2 Munich Institute of Integrated Materials, Energy and Process Engineering (MEP), Technical University of Munich, Garching, Germany; 3 Chair of Bioseparation Engineering, TUM School of Engineering and Design, Technical University of Munich, Garching, Germany; 4 Chair of Renewable and Sustainable Energy Systems, TUM School of Engineering and Design, Technical University of Munich, Garching, Germany

**Keywords:** alternative protein, bioprocess optimization, cellular agriculture, cultured meat, media recycling, sustainable bioprocessing, cell-based food, cultivated meat

## Abstract

**Introduction:**

Despite growing interest in cultured meat, scaling its production from laboratory systems remains challenging. Its industrial translation is constrained by a lack of process-level models and validated engineering data.

**Methods:**

In this study, we developed a process model for animal cell biomass production in suspension batch culture using SuperPro Designer v12. The model describes suspension-based cell proliferation to produce unstructured biomass without microcarriers and excludes cell differentiation, tissue maturation, or structured tissue formation. It integrates media preparation, sequential cell expansion up to a 20,000 L stirred-tank reactor, downstream clarification and washing, extrusion, and supporting utilities into a single flowsheet. Unit operations were parameterized using literature-derived assumptions from mammalian suspension culture, enabling complete mass and energy balances throughout the system.

**Results:**

The baseline model assumes a maximum cell density of 5 × 10^7^ cells/mL, and yields ~2,000 kg of biomass per 30.7-day batch. Media preparation and sterilization, cleaning-in-place- and cooling-related utility demand, and cell expansion performance emerged as major scale-up bottlenecks. Scale-out simulation revealed disproportionally high utility and cultivation time demands relative to production output. Waste and utility streams, primarily depleted medium, cleaning-in-place effluents, and cooling demand, emerge as critical targets for sustainability assessment and valorization.

**Discussion:**

The model is not intended as a final process design but as a reference framework: it makes assumptions explicit, provides reproducible balances, and supports comparative scenario analysis. This framework enables systematic evaluation of scalable cultivated biomass production concepts, highlights technical potential and infrastructure requirements, and helps prevent misdirected investments by allowing process configurations to be assessed before industrial implementation.

## 1 Introduction

Cultured meat (CM), also referred to as cultivated or cell-based meat, is produced by expanding animal cells ex vivo in controlled bioreactor environments. It has been proposed as a technology capable of reconciling consumer demand for meat with environmental and ethical imperatives (1, 2). Since the first public demonstration of a cultured beef burger in 2013, the field has made considerable advances in cell line development, serum-free media, and bioreactor technologies. However, industrial-scale implementation remains constrained by the absence of validated process models and the scarcity of techno-economic and life-cycle data (3, 4). Existing assessments often rely on highly simplified assumptions and extrapolations from biopharmaceutical processes, which are ill-suited to capture CM’s unique features. In particular, nutrient demand at high cell densities, downstream processing into food-grade biomass, and auxiliary operations such as sterilization and cleaning-in-place (CIP) remain underexplored but are critically important in determining cost and sustainability (5, 6). A robust, mechanistic process model is necessary to identify cost drivers, quantify waste streams, and provide a transparent basis for comparing scenarios. Here, we present a comprehensive process model for industrial-scale cultivated animal cell biomass production developed in SuperPro Designer v12. The model describes suspension-based cell proliferation for the production of unstructured biomass without microcarriers and does not account for cell differentiation, tissue maturation, or structured tissue formation. Although the long-term vision in the cultured meat industry is the production of structured whole-cut products that replicate the texture, taste, and sensory properties in general of conventional meat (2), unstructured biomass products represent a more realistic near-term commercialization target due to their lower biological and technological complexity. Several authors suggested that early products will likely resemble processed meat products such as nuggets, sausages, burger patties, meatballs, and other minced meat-style foods (3, 7, 8). The exclusion of differentiation and tissue maturation simplifies the modeled process and inevitably leads to lower production time, energy, utilities, materials, and infrastructure demand, as well as lower process complexity, compared to producing structured cultured meat products. The model therefore integrates media preparation, multi-stage cell expansion, and downstream processing to produce a ground-meat-like product while explicitly resolving mass and energy balances. By combining published formulations with carefully derived assumptions, the model provides a structured framework for techno-economic and sustainability assessments, highlighting critical parameters for industrial translation.

## 2 Materials and methods

All process simulations were conducted in SuperPro Designer v12 (Intelligen Inc., USA), following the standard methodologies in process systems engineering as outlined by Petrides et al. (9). Each unit operation was parameterized based on literature values, vendor specifications, and assumptions where empirical data were unavailable. Where relevant, default components from the SuperPro Designer database were replaced with user-defined pseudo-components (e.g., proteins, growth factors, depleted medium) to reflect CM-specific process streams.

**Figure 1:**
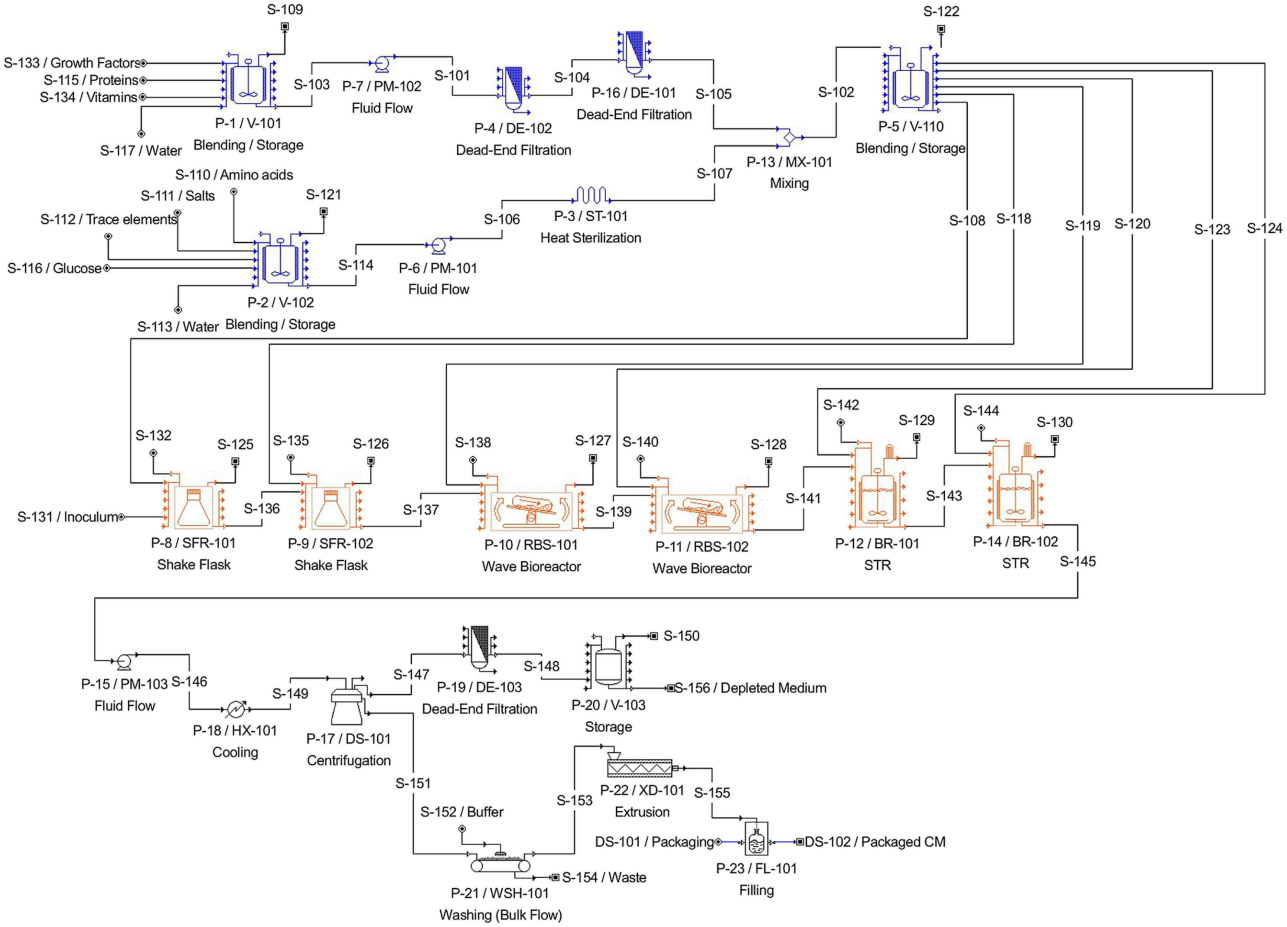
Full process model for industrial production of animal cell biomass. The full process contains three parts (from top to bottom): media preparation from blending tanks V-101 and V-102 to storage tank V-110, cell expansion with initial shake flask SFR-101 up to final 20,000 L stirred-tank reactor BR-102, and downstream processing resulting in a product stream DS-102 and waste-streams “Depleted Medium” S-156 and “Waste” S-154.

The modeled process, displayed in Figure 1, was defined to include (i) media formulation and sterilization, (ii) inoculum thawing and sequential expansion to a final stirred-tank reactor (STR), (iii) downstream separation, washing, extrusion, and packaging, and (iv) auxiliary operations such as sterilization-in-place (SIP) and cleaning-in-place (CIP), representing a gate-to-gate production process within the facility.

To enable reproducibility, several baseline assumptions were established: cells were cultured in suspension without microcarriers, the product was modeled as unstructured, ground-meat-like biomass, and the production scheme followed a batch operation with in-house media preparation and product formulation. Although fed-batch supply of media is favored in large-scale CM production (5), which ensures constant concentration of nutrients and higher achievable substrate concentrations (10), batch operation was selected for the model to simplify the scheduling of different operations and only focus on the mass and energy balances. Since no medium is removed in either batch or fed-batch process, this simplification did not change the medium requirement. Furthermore, fed-batch supply of fresh medium does not prevent the accumulation of toxic metabolites (10) because no additional output streams are present, compared to the batch mode of operation. Therefore, all input and output streams, along with their respective compositions, remain valid under this simplification. This is not true for perfusion systems, which require a different model with a continuous supply of fresh medium and the removal of spent medium along with the product (10). Therefore, the presented model can be adapted to feature a fed-batch approach with additional data needed to gain valuable insights on the expected differences between batch and fed-batch approaches. These include but are not limited to: (i) a better understanding of concentration-dependent growth kinetics for the specific cell culture, (ii) validated real-life equipment data regarding sizing, timing, and energy/utilities demand, and (iii) specific media preparation, storage, and supply strategies. Until then, introducing a fed-batch approach only results in a more difficult process setup with timing differences compared to the current approach, without delivering meaningful insights for a comparative analysis of both approaches.

The model was designed as a generalized suspension-based mammalian cell culture framework rather than a specific proprietary cell line. Suspension cultivation was selected due to its relevance for scalable industrial bioprocessing and compatibility with established stirred tank reactor systems, as well as state of the art in CM (3, 5). Key parameters including doubling time, inoculation density, and maximum cell density were derived from published mammalian suspension culture literature and techno-economic assessments rather than arbitrarily assumed (3, 5, 11). They are summarized in Table 1, together with their individual literature reference, and the corresponding uncertainty ranges. The maximum doubling time was assumed to be 20 h based on the values reported for CHO-K1 suspension cultures (12). Although different cell lines will likely be used for industrial production of animal cell biomass, it is expected that they will be selected and optimized for high growth rates, making this ambitious assumption feasible even in large-scale systems. Furthermore, the doubling time in this model was only used to check for the overall feasibility. Instead of using a fixed doubling time each reactor was given a fixed timeframe of 5 days (120 h) for cell culturing. The start and end cell counts as well as the number of doubling times based on the 20 h for each reactor are tabulated in Table 2. The calculated values are all below the upper limit of 120 h/20 h = 6 doubling times. Followingly, the model provides room for non-ideal growth rates without limitations to the exact allocation of culturing time per stage. Therefore, its increase due to nutrient gradients and accumulation of metabolic waste, such as lactate and ammonia, during cultivation in the bioreactor was not explicitly modeled. Instead, 20 h represents an average value, assuming a highly optimized cell line and cultivating conditions, while implicitly accounting for the aforementioned effects in an average manner. Inoculation density was 1 × 10^7^ cells/mL, and transfer thresholds were aligned with mammalian culture practice (5). The maximum cell density was set to 5 × 10^7^ cells/mL in the production STR, consistent with the reported density achieved in CHO culture (11). Parameters were cross-validated with mammalian cell culture literature and techno-economic assessments, ensuring plausibility while explicitly highlighting knowledge gaps specific to CM (3).

**Table 1:** Key modeling assumptions and baseline values together with their justification and expected ranges based on available literature data.

Parameter	Baseline value	Expected range	Justification	Reference
Doubling time	20 h	20 h–65 h	Based on already achieved growth rates for mammalian cell culture, assuming that future cell line development will offset the growth rate-decreasing effects of large-scale production. Note: This value was not used in the model, instead every stage was cultured for 5 days and the corresponding doubling times were calculated from the respective start and end cell counts to check for viability with a theoretical doubling time of 20 h (refer to Table 2).	(12, 33)
Cell density in cell bank inoculum	1 × 10^7^ cells/mL	1 × 10^7^ cells/mL	Reported viable cell density in cryovials from cell banking used for production.	(13, 37)
Inoculation cell density	2 × 10^5^ cells/mL (normal process without high cell density inoculation)	2 × 10^5^ cells/mL	Used inoculation cell density for shake flask reactors above the reported minimum (critical) cell density of around 1 × 10^5^ for mammalian cell culturing at industrial scale.	(12, 37)
Maximum cell density	5 × 10^7^ cells/mL	3.5 × 10^6^ cells/mL–8.6 × 10^7^ cells/mL; The maximum reported for animal cell cultures is in the range of 10^8^ cells/mL	Representative value based on reported mammalian suspension culture performance and techno-economic cultivated meat assessments. Assumption requires advancements in high cell density cultivation of animal cells at industrial scale.	(3, 12, 38)
Culture temperature	37 °C	37 °C	Established standard for mammalian cell culturing.	(37)
Maximum biomass concentration	105 g/L	Up to 110 g/L (at 20,000 L working volume)	This assumption requires advancements in high cell density cultivation of animal cells at industrial scale, as cell concentrations around 100 g/L are currently established in bacterial cell cultures.	(3, 38)

**Table 2:** Cell expansion equipment working volumes and cell counts.

Vessel	Working volume	Starting cell count	End cell count	Number of cell doublings
SFR-101	100 mL	2.0 ∗ 10^7^	1.0 ∗ 10^9^	5.64
SFR-102	1,600 mL	1.0 ∗ 10^9^	1.6 ∗ 10^10^	4.00
RBS-101	25 L	1.6 ∗ 10^10^	2.5 ∗ 10^11^	3.97
RBS-102	250 L	2.5 ∗ 10^11^	2.5 ∗ 10^12^	3.32
BR-101	2,000 L	2.5 ∗ 10^12^	2.0 ∗ 10^13^	3.00
BR-102	20,000 L	2.0 ∗ 10^13^	1.0 ∗ 10^15^	5.64

Vessels, as shown in Figure 3, have working volumes. Cell counts calculated with the transfer cell density of 1 × 10^7^ cells/mL and final stage cell density in BR-102 of 5 × 10^7^ cells/mL. Number of doubling times calculated with Equation (1).

The methodology used in biopharmaceutical modeling was adapted here for the production of animal cell biomass. Rather than representing a single proprietary process, the model establishes a reference flowsheet with transparent assumptions, enabling reproducibility and comparative analysis. The model was designed to resolve whole-mass and energy balances, enabling a quantitative assessment of resource inputs, product formation, waste streams, and energy demands. These assessments were performed for four simulated scenarios to compare different production strategies:

- Scenario 1: Main Process, 90:10 split, in which 90% of the medium volume was sterilized by sterile filtration and 10% by heat sterilization during media preparation.
- Scenario 2: Main Process, 50:50 split, in which the medium volume was equally divided between sterile filtration and heat sterilization during media preparation.
- Scenario 3: Local Scale Variation, representing a decentralized, smaller-scale production process derived from Scenario 1, with a final cell expansion working volume of 2,000 L and downstream processing limited to biomass washing and cooling rather than extrusion and filling.
- Scenario 4: Cell Expansion Variation, representing a modified upstream process based on high cell density cryopreservation and five stirred tank reactors instead of the original six-stage expansion with shake flasks, wave bioreactors, and stirred tank reactors.

### 2.1 Main process

In the main process, 2 mL of the starting culture (cryostock) was used (13). In the final stage, cells were expanded to a STR with a working volume of 20,000 L (3). Two different scenarios of the main process were simulated: *Scenario 1: Main Process, 90:10 split,* and *Scenario 2: Main Process, 50:50 split*. The respective simulation files are both contained in the Supplementary Materials. They refer to the two split options between sterile filtration and heat sterilization during media preparation of 90:10 and 50:50. In media preparation, the sterilization is of key importance to ensure product safety and prevent the intake of unwanted/harmful organisms into the fermentation stage. While certain components (vitamins, proteins, and growth factors) are heat-sensitive and must be sterilized via filtration, water constitutes a large fraction of the medium and can be sterilized using either method. It was decided to present two options for media sterilization, differing in the distribution of medium broth between the two sterilization methods. For the 90:10 split, 90% of the total medium broth volume was subjected to sterile filtration and only 10% to heat sterilization. In contrast, for the 50:50 split, the shares were equal, with 50% of the total volume sterilized via filtration and 50% via heat. The two options allow for later highlighting the difficulties in assessing different process options from a sustainability viewpoint, as each method has certain advantages and disadvantages that can lead to distinct conclusions based on the specific plant design, plant environment, and sustainability goals. This is discussed in detail in sections 3.1 and 4. Differences between the two files regarding the unit procedures and their operations were only present for the media preparation stage. The cell expansion and downstream processing stages were identical, except for minor scheduling differences caused by differing durations of the cooling operation in V-110.

#### 2.1.1 Media preparation

**Figure 2:**
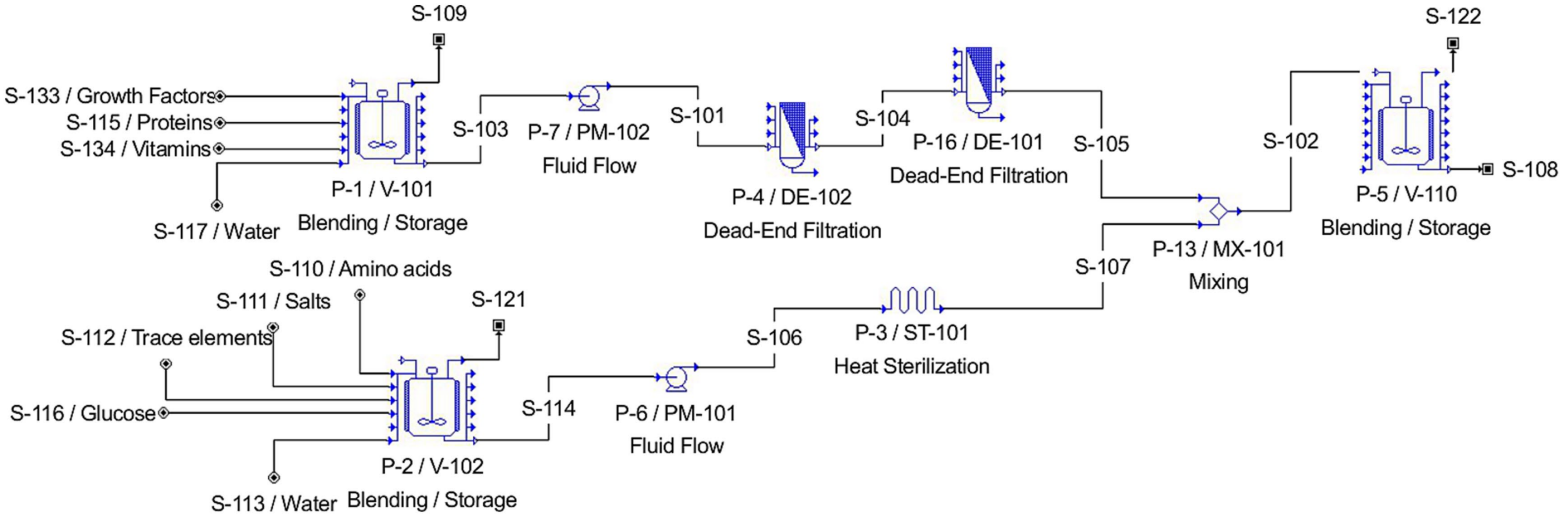
Visual representation of media preparation obtained from SuperPro Designer. Heat-sensitive components (growth factors, proteins, and vitamins) were sterilized by dead-end filtration, while others (trace elements, salts, amino acids, and glucose) were heat sterilized. Both streams were mixed in MX-101 and stored as a single component “Medium” at 4 °C.

For media preparation, it was decided to rely on a two-way system, utilizing heat sterilization for heat-resistant media components and sterile filtration for temperature-sensitive components. The components were categorized under the following headings: amino acids, growth factors, proteins, salts, trace elements, and vitamins. These six were added as components based on water properties and set as “Solid or Non-Volatile” with 100% dry mass content. Additionally, glucose was added as a component available in the SuperPro Designer database. These seven components were mixed with water in two blending tanks, where unit procedure P-1, associated with equipment V-101, contains the growth factor, protein, and vitamin streams for sterile filtration, and unit procedure P-2, associated with equipment V-102, includes the amino acid, glucose, salt, and trace element streams for heat sterilization. After blending, the tanks were emptied by the respective centrifugal pumps P-7 / PM-102 and P-6 / PM-101 before entering either the heat sterilization unit procedure P-3 with ST-101 or the two-stage filtration comprised of dead-end filtrations P-4 / DE-102 and P-16 / DE-101. The two sterilized streams were then guided through a mixing unit P-13 / MX-101 before entering the storage tank P-5 / V110. The process scheme is also visualized in Figure 2.

To model the media preparation, a dummy media solution had to be created. As no information on the cell culture media used in current industrial CM processes is publicly available, an artificial medium was developed based on the compositions of commercially available animal cell culture media. By combining four common animal cell culture media available for purchase—AdvancedMEM (14), RPMI 1640 (15), DMEM (high glucose) (16), and IMDM (17), an artificial medium was created. Its formulation is given in Table 3. This approach was selected to avoid bias toward a specific medium while ensuring that all necessary components are available in sufficient amounts for high-cell-density cultures. For amino acid, vitamin, and salt content, the concentrations were calculated as the arithmetic means of the four media. Trace elements were only tabulated in the source data for AdvancedMEM, so this value was adopted. Trace metals are commonly provided at sub-micromolar concentrations, whereas sodium, potassium, magnesium, and calcium ions are included as salts (18). The glucose content was assumed to be high, given that high cell densities are desired in the process; therefore, the value from high-glucose DMEM (4.5 g/L) was chosen. The media are supplemented with fetal bovine serum (FBS). These animal-derived products provide additional essential nutrients, such as hormones and growth factors, that are not present in the medium formulation. Typically, they are added before use and account for approximately 5 to 10% of the total medium solution (19–21). Proteins, of which growth factors are a subtype, are crucial in animal cell culture and are therefore added as a category of medium components. According to product specifications, FBS contains 30–40 g/L of total protein. However, measurements have shown values of around 22.85 g/L and 33.5 g/L (19). Therefore, the lower specification limit of 30 g/L is chosen as the reference for protein content (22–24). For growth factor concentration, values for IGF-1 content of around 46 mg/L and 70 mg/L in FBS were measured, while other growth factors were measured at even lower concentrations in the tens of ng/L region, so the IGF-1 content was used as a reference at 50 mg/L (23, 25). Assuming the substitution of 10% FBS addition, the protein and growth factor concentrations were divided by 10, while the other concentrations derived from the media compositions were assumed to stay undiluted. To calculate the total demand for each component per batch, the total medium demand, which corresponds to the final working volume of 20,000 L, was multiplied by the respective concentrations. The results are tabulated in Table 3. These amounts of the components were added to the respective inlet streams, and the amount of water was adjusted such that, after mixing in P-13 / MX-101, a total medium volume of 20,000 L was obtained.

**Table 3:** Composition of the artificial medium used for the production of animal cell biomass and the per-batch-amount of each component.

Components	Concentration in g/L	Amount in kg/batch
Total solids	18.40	367.91
Amino acids	1.27	25.44
Vitamins	2.94 ∗ 10^−2^	5.87 ∗ 10^−1^
Salts	9.59	191.78
Proteins	3.00	60.00
Trace elements	5.01 ∗ 10^−3^	1.00 ∗ 10^−1^
Glucose	4.50	90.00
Growth factors (IGF-1)	5.00 ∗ 10^−6^	1.00 ∗ 10^−4^

To calculate the total demand for each component per batch, the total medium demand, which corresponds to the final working volume of 20,000 L, was multiplied by the respective concentrations.

The most important settings are outlined below. In scenarios 1 and 2, components were mixed in the first tanks for 60 min at a specific power of 0.1 kW/m^3^, which was the fallback value. For both scenarios, the following “Transfer Out” operation, during which agitation was continued, was set to last approximately 5 h. For the 50:50 split, with 10,000 L each, the flow rate was set to 2000 L/h. For the 90:10 split, the flow rate for the 18,000 L in V-101, intended for sterile filtration, was set to 3,600 L/h. For the 2,000 L in V-102, used for heat sterilization, the flow rate was 400 L/h. The pumps were set to follow this timing and increase the pressure head by 1 bar to 2.013 bar absolute pressure. The temperature was maintained at 25 °C up to this point. For sterile filtration, the growth factor, protein, and vitamin streams were contaminated with an “Impurities” component at 0.1% of the mass of the respective medium component, before entering the blending vessel in P-1. These impurities were filtered in P-4 at 80% and then 100% in P-16; the filtration flux was maintained at the fallback value of 250 L/m^2^∙h. For heat sterilization, the sterilization criterion was set to the fallback value, while the sterilization temperature was maintained at 121 °C (or 250 °F), a standard steam sterilization temperature (26–28). Steam at 152 °C was used as the heating agent. At the same time, water at 25 °C inlet temperature and 30 °C outlet temperature served as the cooling agent, resulting in a final exit temperature of 35 °C for the sterilized medium. The heat exchange agent streams are not visible in the process scheme. The streams from sterile filtration and heat sterilization were then mixed in P-13 and entered the storage unit V-110. In the tank, the components reacted to form a single component, “Medium,” based on water, according to mass-conserving Reaction (1), which was stored at 4 °C and 1.013 bar.

$$\begin{array}{l} 25.4375\;\text{Amino Acids}+90.0000\;\text{Glucose}\\ +0.0001\;\mathrm{IGF}-1\\ +60.0000\;\text{Proteins}+191.7802\;\text{Salts}\\ +0.1001\;\text{Trace Elements}+0.5874\;\text{Vitamins}\\ +19539.6850\;\text{Water}\to 19907.5903\;\text{Medium} \end{array}$$

Reaction (1)

#### 2.1.2 Cell expansion

**Figure 3:**
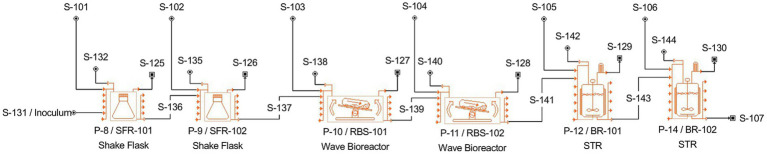
Visual representation of the cell expansion obtained from SuperPro Designer. It includes shake flasks for inoculation, wave bioreactors for intermediate scale-up, and stirred-tank reactors. The working volume increased with each vessel from 100 mL to 20,000 L, as shown in Table 2. After the creation of biomass according to Reaction 2 in each unit operation, the content was emptied into the next vessel as inoculum until leaving the final stage BR-102.

For cell expansion, a classic three-stage approach including shake flasks, wave bioreactors, and STRs was chosen. In each stage, two vessels of increasing capacity were used, so that the working volume increased approximately tenfold from one vessel to the next, with the exception of the first stage, in which shaking flasks were used. In total, the process comprises six-unit procedures and vessels for cell expansion. These are, in order, P-8 with a 125 mL shake flask, SFR-101, P-9 with a 2 L shake flask, SFR-102, P- 10 with a 50 L wave bioreactor RBS-101, P-11 with a 500 L wave bioreactor RBS-102, P-12 with a 2,500 L STR BR-101, and finally P-14 with a 25,000 L STR BR-102 as depicted in Figure 3. For storage tanks, only an air outlet for venting needs to be included, as the inlet is automatically presumed by the software without a visible stream, as seen in Figure 2 in Section 2.1.1. In contrast, bioreactors are required to have both a designated inlet and outlet stream. Apart from these air supply and venting streams, every procedure has two inlet streams: on the one hand, the inoculum stream, starting with S-131 “Inoculum” at P-8 and then continuing from vessel to vessel, and on the other hand, the medium supply stream originating at P-5 / V-110 – coming in from the top in Figure 3. After cultivation, the outlet streams transferred the contents of each vessel to the next stage of inoculum expansion. The final outlet stream, S-145, served as the starting point for downstream processing.

For the shake flasks and STRs, the maximum working volume was assumed to be 80% of the vessel volume. For the wave bioreactors, the maximum working volume was set at 50% of the total volume. The following working volumes for culturing in each vessel, assumed for the modeling, are tabulated in Table 2. The “Inoculum” stream was modeled simply as 2 mL of “Biomass,” a component readily available in the SuperPro Designer database. The number of doubling times 𝑁 was calculated with the following Equation (1), and the results are listed below in Table 2:

$$N=\log_{2}(\frac{\mathrm{end}\;\text{cell count}}{\text{starting cell count}}).$$ (1)

The resulting number of doubling times was below 6 for all vessels, and with the assumed doubling time of 20 h, the calculated culturing times ranged from 60 h to 112.8 h. It was decided to use a culturing time of 5 days (120 h) or 6 doubling times per vessel. Secondly, the amount of fresh medium added in each expansion stage was determined by subtracting the inoculum volume (working volume of the previous vessel) from the working volume of the respective vessel. This approach assumes a volume-preserving, batchwise fermentation, in which the culture is expanded to a final working volume of 20,000 L in reactor BR-102. Based on these considerations, the operations inside the unit procedures were set up as follows:

1. Operation “Purge-1”: Pressure purging at 1.5 bar and 25 °C with mixture “Synthetic Air.”
2. before returning to the reference pressure of 1.013 bar.
3. Operations “Transfer In-1” and “Transfer In-2”: Loading with the medium from V-110 and the contents (non-gaseous) of the previous reactor – for the first shake flask SFR-101, the “Inoculum” stream is instead loaded via a “Charge” operation.
4. Operation “Ferment-1”: Stoichiometric batch fermentation at 37 °C for 5 days, aeration with “Synthetic Air” and venting through the respective gas streams, conversion according to Reaction (2).
5. Operation “Transfer Out-1”: Transferring the non-gaseous content of the vessel out at a flow rate in L/min consistent with the working volume, such that roughly 1 min is needed – except for BR-102, where the flow rate was set to 4,000 L/h.

The “Synthetic Air” mixture was comprised of 5% CO_2_, added from the SuperPro Designer database, and 95% “Air,” a predefined mixture of 79% N_2_ and 21% O_2_ (percentages refer to moles). The loading of the vessels with the fermentation broth from the previous reactor followed the transfer-out operation, thereby taking a negligible amount of time. The loading with medium was set at different flow rates via “Split” operations, transferring the “Medium” component from P-5 / V-110, thereby achieving increased loading times that varied depending on the amount, ranging from minutes to hours. These loading operations were scheduled to be completed with the inoculum loading. The amount of medium transferred into each vessel differs from previous considerations, as thermal contraction between the storage temperature of 4 °C and the initial temperature of 25 °C or the fermentation temperature of 37 °C is not negligible. “Split” operations require a mass, not a volume, to be transferred. The masses were calculated as the ratio between the medium requirement for the respective vessel and the total medium demand of 20,000 L multiplied by the actual amount of the “Medium” component stored in V-110 (19,907.59 kg). This gives, for example, 97.55 g for SFR-101. For the fermentation operation, numerous parameters had to be set, most of those were left at the fallback values, such as aeration rate and power consumption for agitation. The agitation energy was intended to be converted entirely into heat within the reactors, necessitating the use of a cooling agent to maintain a constant temperature of 37 °C. “Chilled Water” at an inlet temperature of 5 °C and outlet temperature of 10 °C was chosen. The fermentation itself was modeled simply as a batch operation with a stoichiometric reaction. The stoichiometric reaction takes “Medium” and “Oxygen” (O_2_). It transforms them into the components “Biomass,” “Carbon Dioxide” (CO_2_), and “Impurities” (all previously introduced), as well as “Depleted Medium,” which is set up based on water properties, according to the ratios presented in Reaction (2). The reaction was set to achieve 100% conversion without generating heat. 100% conversion does not imply that all the nutrients from the medium were taken up by cells and converted to biomass. Instead, “Depleted Medium” contains residual amounts of these nutrients, which are not modeled explicitly. Although calorimetric experiments show that animal cell growth is slightly exothermic, this contribution was neglected, as it is relatively small, and heat is commonly lost to the surroundings faster than it is generated in the culture (3). Venting ensured a constant pressure of 1.013 bar. Due to greater CO_2_ generation than O_2_ consumption, the fermentation reaction results in a slight overall loss of material into the gaseous phase. Therefore, the process was not volume-preserving for the liquid phase, in contrast to previous assumptions.

$\begin{array}{l} 100\;\text{Medium}+5\;O_{2}\to 5\;\text{Impurities}+10\;\text{Biomass}\\ +10\;{\mathrm{CO}}_{2}+80\;\text{Depleted Medium} \end{array}$ Reaction (2)

#### 2.1.3 Downstream processing

**Figure 4:**
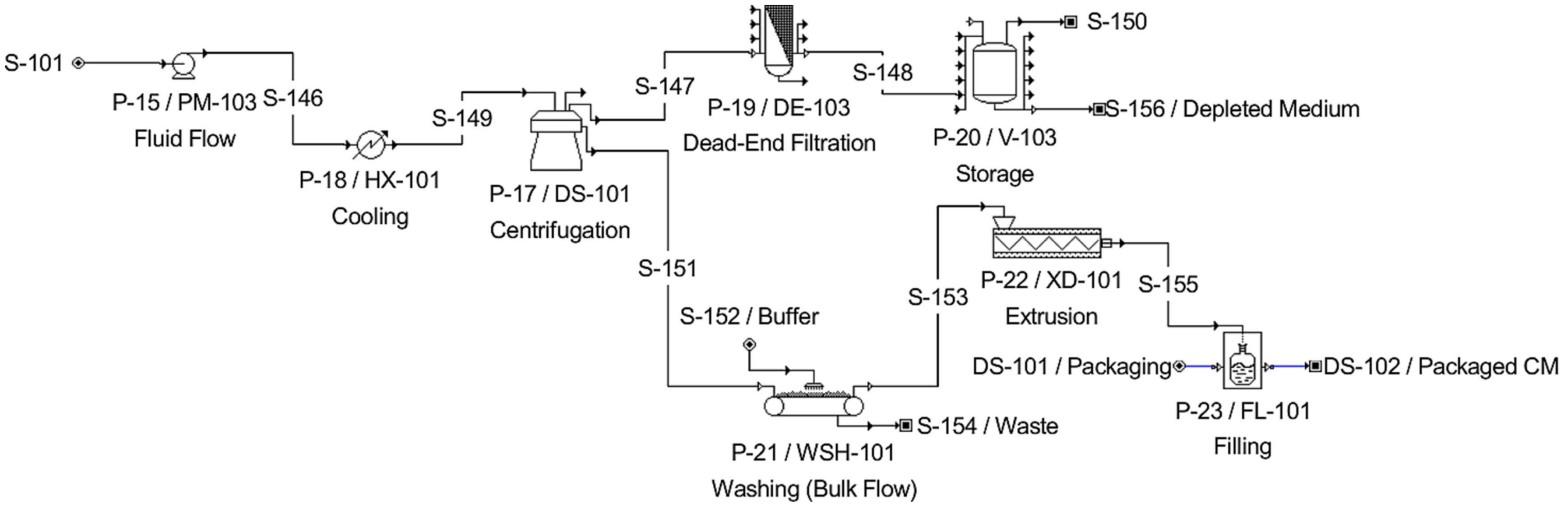
Visual representation of the downstream processing obtained from SuperPro Designer. After the initial cooling, the solid–liquid separation in centrifuge DS-101 created two outlets where S-147 was mostly comprised of liquid “Depleted Medium” and S-151 mostly of solid “Biomass.” Stream S-151 was further processed in washing, extrusion, and packaging operations.

Downstream processing begins with the BR-102 outlet stream, whose composition is presented in Table 4. The stream has a temperature of 37 °C at 1.013 bar and is denoted as S-145. The material stream entered the pump P-15 / PM-103 and was guided through a cooling device represented by P-18 / HX-101 before entering the disk stack centrifuge P-17 / DS-101. The centrifuge created two outlet streams, where S-147 represents the waste stream with “Impurities” and “Depleted Medium,” which was guided through a dead-end filtration for sterilization in P-19 / DE-103 before entering the storage tank P-20 / V-103 and finally reaching outlet stream S-156 “Depleted Medium.” Product-containing stream S-151 was guided through a washing device, P-21 / WSH-101, where another inlet stream, S-152 “Buffer,” was introduced, and again two outlet streams, S-154 and S-153, were created. The first contained waste and was not subjected to another unit procedure. The product-containing stream S-153 was guided into an extrusion unit procedure, P-22 / XD-101, and subsequently into unit procedure P-23 / FL-101, where it was combined with stream DS-101, “Packaging,” in a filling process, thereby turning the bulk stream into a discrete stream. The packaged product was leaving the process as DS- 102. The entire downstream process is depicted in Figure 4.

**Table 4:** Composition of stream S-145 leaving the cell expansion stage.

Component	Amount in metric tonnes	Mass% of component
Biomass	1.99	10.53
Depleted Medium	15.93	84.21
Impurities	1.00	5.26

S-145 leaves the unit procedure P-14 / BR-102 toward downstream processing. The stream’s temperature is 37.0 °C at 1.013 bar.

The “Transfer Out” operation of BR-102 was set with a volumetric flow rate of 4,000 L/h. The pump at P-15 followed this operation and again increased the pressure by 1 bar. The timing of the entire downstream process was set to depend on this “Transfer Out” operation. The unit procedures all contained one operation to manipulate the respective stream(s), except for P-20, which included a “Transfer In” and a “Transfer Out” operation, often followed by a CIP operation, as described in Section 2.1.4. The cooling at P-18 utilized chilled water to reduce the stream temperature to 25 °C. The centrifuge operation of P-17 was set to “solids removal” with 95% “Biomass” removal, 5% “Impurities” removal, and a particulate concentration of 400 g/L for the solids stream. At the same time, other settings were left at fallback values. Additionally, the centrifuge operation was combined with cooling the stream down to 15 °C, utilizing chilled water again. Stream S-151 represents the solids stream, which contains most of the biomass, whereas stream S-147 contains mostly “Depleted Medium.” The latter was guided through P-19, set to filter components “Biomass” and “Impurities” at 100% removal, before being transferred into P-20 / V-103. To include the CIP of this tank, the entire content was transferred to stream S-156 at 1.013 bar and 15 °C. The washing unit procedure P-21 took S-151 as an input and introduced a new component “Buffer Solution,” based on the properties of water, via S-152. In the “Wash” operation, 2,000 L of “Buffer Solution” at 4 °C (stream S-152) was used to remove 100% of both “Depleted Medium” and “Impurities.” The product-containing stream after the “Wash” operation comprised 10% “Buffer Solution” and 90% “Biomass.” Thermally, the operation was set to approach equilibrium between S-152 and S-151 at 50% efficiency. The waste outlet stream is denoted S-154 “Waste.” The product-containing stream S-153 continued to P-22. The “Extrude” operation of P-22 was set up such that the angular screw velocity was left at the 200 rpm (revolutions per minute) fallback value. In this step, biomass could be conceptually mixed with additives, such as plant proteins, and compacted, for example, using a screw press with a static end plate with holes. However, the addition of such additives was not explicitly modeled and the only manipulation of the stream was due to the inclusion of cooling to 4 °C using the cooling agent with an inlet temperature of −4 °C and an outlet temperature of −3 °C. Therefore, the “Extrude” operation in this model serves only as a placeholder for a downstream processing step in the production. For the filling unit operation P-23, another component, “Container,” based on water properties and set as a solid with 100% dry mass content, was introduced via the discrete stream DS-101. The “Fill” operation packaged 1 kg of the stream S-155 with 10 g of the “Container” component. The resulting entities left FL-101 via stream DS-102, containing the respective amount of product entities with a mass of 1.01 kg each at 4 °C.

#### 2.1.4 CIP operations

Generally, CIP operations were incorporated into the unit procedures. The standard CIP templates provided by the software SuperPro Designer have been adapted for use in the CM production process, as follows. Two templates were created: one extensive CIP protocol for storage tanks and bioreactors, where material streams have considerable retention times, and one reduced CIP protocol for flow-through unit operations and equipment, such as pumps, centrifuges, and others. The full templates for both protocols are provided in the Supplementary Materials. The first one was based on the “Product Vessel CIP Cycle”, which includes five steps. Henceforth, it shall be known as “CIP Template_Full.”

It was applied to the following unit procedures / equipment: P-1 / V-101, P-2 / V- 102, P-5 / V-110, P12 / BR-101, P14 / BR-102, P-20 / V-103. The CIP operation was added after the last operation of the actual production process in all cases. For the tanks (P-1, P-2, P-5, and P-20), a “Purge” operation was added after CIP, during which the vessels were pressure filled with “Air” for 30 min at 1.5 bar before returning to the reference pressure of 1.013 bar. For the bioreactors, this was omitted because they are purged before fermentation. The second, reduced template, only involves three steps and was applied to the following unit procedures and/or equipment: P-7 / PM-102, P-6 / PM-101, P-3 / ST-101, P-15 / PM-103, P-18 / HX-101, P-17 / DS-101, P-21 / WSH-101, P-22 / XD-101, P-23 / FL-101. Since the type of equipment and, subsequently, the specific characteristic dimension regarding size, differ between these unit procedures, no characteristic dimension-specific flow rate was used compared to “CIP Template_Full.” Instead, the volumetric flow rate was set to the nearest full hundred L/h to the throughput during the production process for the respective unit procedure. Some unit procedures were left without CIP operations. For the shake flasks and wave bioreactors, no CIP operation can be added in SuperPro Designer software, and the use of autoclaves was not modeled. For the dead-end filtrations (DE-101, DE-102, DE-103) and the mixing (MX-101), it was omitted. Three washing solutions, Acidic Wash Solution (AWS), Caustic Wash Solution (CWS), and Sanitizing Wash Solution (SWS), were added as mixtures of “Water” and another active ingredient. These active reagents were registered as components from the SuperPro Designer database. The compositions of the solutions are given in Table 5.

**Table 5:** Compositions of washing solutions used for cleaning-in-place operations.

Washing solution	Full name	Active component	CASRN of component	Mass% of component
AWS	Acidic Wash Solution	Nitric Acid (HNO_3_)	7,697-37-2	0.5
CWS	Caustic Wash Solution	Sodium Hydroxide (NaOH)	1,310-73-2	1.0
SWS	Sanitizing Wash Solution	Sodium Hypochlorite (NaClO)	7,681-52-9	1.0

### 2.2 Additional process variations

This section describes additional modeling and simulation approaches. They are based on the primary production process and its subprocesses from Section 2.1. The files are also included in the Supplementary Materials. First, an additional production process for local scales is introduced as *Scenario 3: Local-Scale Variation*. Then, the subdivision of the production process into three independent files, covering the three stages of media preparation, cell expansion, and downstream processing, is briefly described before introducing a variation of the cell expansion stage, *Scenario 4: Cell Expansion Variation*.

#### 2.2.1 Scenario 3: local-scale variation

A local-scale production process suitable for decentralized production of animal cell biomass was modeled and compared with the main process. It was created by copying and adapting the production process from Section 2.1., *Scenario 1: Main Process, 90:10 split*. In the first step, P-14 / BR-102 was removed, leaving P-12 / BR-101, with a vessel volume of 2,500 L, as the final stage of cell expansion. Consequently, the maximum working volume was 2,000 L, or one-tenth of the main process. The cell expansion stage was otherwise left untouched. The amount of required medium was subsequently reduced to 2,000 L, necessitating an adjustment to the media preparation stage. The inlet streams were adjusted to the new, per-batch amounts of the respective components, according to the artificial medium composition. The outlet flow rates for mixing tanks P-1 / V-101 and P-2 / V-102 were reduced to a tenth of the original values, with 360 L/h and 40 L/h, thereby affecting the following pumps and sterilization unit procedures up to tank P-5 / V110. The required time for the media preparation was thereby kept the same as in the primary production process. For heat sterilization in P-3 / ST-101, an adjustment of the fluid flow’s Reynolds number from 10,000 to 9,500 was necessary, as the simulation would not converge with the previous setting. The adjustment is considered negligible in relation to the subsequent process analysis. The amount of medium transferred into each of the five bioreactors from V-110 via “Split” operations was again calculated as described in Section 2.1.2. For the stream S-124 from P-12 / BR-101 (cell expansion) to P-15 / PM-103 (downstream processing), the flow rate of the respective “Transfer Out” operation was reduced by a factor of 10 to 400 L/h, thereby maintaining the DSP time consistent with the primary process. The downstream process was reduced by removing the extrusion P-22 / XD-101 and the filling P-23 / FL-101. The idea was that, for local-scale production, the product is only prepared for further processing at a plant that receives raw cultured meat from multiple plants. Therefore, only the “Depleted Medium” and “Impurities” removal, including P-21 / WSH-101, were retained after centrifugation. Instead, an electric cooling unit procedure (P-14 / EC-101) was added after washing, cooling the product-containing stream to 4 °C. The product-containing stream terminates with S-143 after this unit procedure. Other settings and the waste-containing stream after centrifugation in the downstream processing section were not altered from the main process. Lastly, CIP operations had to be adapted. While “CIP Template_Full” scales with the equipment sizes determined by the software, the reduced template has manually set flow rates, which are based on the respective unit procedures’ throughputs. These were again reduced by a factor of 10 compared to the original process. The reduced CIP was also added for P-21 / WSH-101. Again, the shake flasks, wave reactors, dead-end filtrations, and mixing procedure P-13 / MX-101 were left without a CIP operation.

#### 2.2.2 Subdivision of the main process

To facilitate the investigation and variation of the subprocesses, the model for Scenario 1: Main Process, 90:10 split was adapted into standalone models, ready for simulation. Scenario 1 was selected because it represents the baseline process configuration of this study, whereas Scenario 2 was introduced specifically as a comparative variation of the sterilization strategy during media preparation. Since both scenarios only differ in the volumetric split between sterile filtration and heat sterilization, while cell expansion and downstream processing remain unchanged apart from minor scheduling effects, Scenario 1 provided the most suitable reference case for the standalone investigation of the individual subprocesses. This choice also ensured consistency with the subsequent process variations, which were likewise derived from the 90:10 baseline. Additional necessary adjustments were as follows:

- For the media preparation, the 90:10 split ratio was used. The “Split” operations transferring medium to the bioreactors were removed from P-5 / V-110 and replaced with a single “Transfer Out” operation into the added outlet stream S-108.
- For the cell expansion, the medium addition was reworked to six single inlet streams, S-101 through S-106, one for each bioreactor. Instead of working with the calculated medium masses used in the total process model, the theoretically needed medium volume (without accounting for the volume loss due to the fermentation reaction) was added at 37 °C for each vessel, e.g., 98 mL for SFR-101. The medium supply via inlet streams required the conversion of the respective “Transfer In” operations into “Charge” operations. The timing for all charging and transferring operations was also lowered to 1 s. The outlet from BR-102 was changed to stream S-107.
- For the downstream processing, only the inlet stream, formerly S-145, had to be recreated. The inlet was set to S-101 with a composition corresponding to the values listed in Table 4.

#### 2.2.3 Scenario 4: cell expansion variation

**Figure 5:**
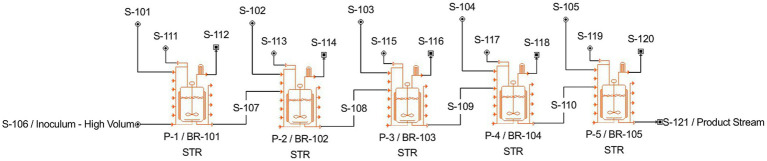
Visual representation of *Scenario 4: Cell Expansion* Var*iation* with high volume inoculum obtained from SuperPro Designer. Compared to the main process (Figure 2), only five stirred-tank bioreactors were used, scaling from 2 L to 20,000 L. The use of high cell density cryopreservation was assumed.

To account for variation in the cell expansion stage, two concepts were introduced. Firstly, the use of a higher volume first-stage inoculum allows for a larger starting volume in fermentation within a bioreactor. This forward-looking concept is known as high cell density cryopreservation (13). Although it is not yet established in industry, it could play an important role in the future of CM production, provided the strategy turns out technically feasible and reliable also in large bioreactors. Secondly, shake flasks and wave reactors were replaced by STRs, eliminating single-use reactors (wave bioreactors) and manual-use reactors (shake flasks). The model was set up based on the standalone cell expansion file as described in Section 2.2.2. Therefore, the medium was introduced via single streams at 37 °C, in accordance with volumetric considerations. The time for charging and transferring operations was set as 1 s. For all bioreactors, purging operations with “Synthetic Air” were added before fermentation, and “CIP Template_Full” was carried out after fermentation. The starting inoculum volume was set as 200 mL of “Biomass” in stream S-106. Five STRs were added, P-1 / BR-101 through P-5 / BR-105, with a tenfold volume increase each step. Vessel/working volume and medium inflow of each STR are listed in Table 6. The fermentation reaction was set to Reaction (2) and lasted 5 days. Other settings were also copied from the main process (see Section 2.1). The “Product Stream” leaves BR-105 as S-121. The process is shown in Figure 5.

**Table 6:** Bioreactor sizing and amount of medium supply for *Scenario 4: Cell Expansion Variation*.

Vessel	Working volume in L	Vessel volume in L	Inlet stream of medium in L
BR-101	2	2.5	1.8
BR-102	20	25	18
BR-103	200	250	180
BR-104	2,000	2,500	1,800
BR-105	20,000	25,000	18,000

In the variation, only STRs were used, and the number of bioreactors was reduced from six to five due to a higher inoculum volume.

## 3 Results

This section concerns the results from the modeling and the subsequent simulation of the modeled production processes. Section 3.1. refers to the main process (Scenarios 1 & 2) presented in Section 2.1. Section 3.2. presents the results from additional modeling variations (Scenarios 3 & 4) as described in Section 2.2. Additional data can be obtained from the reports created with the software, which are included in the Supplementary Materials. SuperPro Designer files for all scenarios are also included in the Supplementary Materials, where all settings, streams, and their compositions can be inspected.

### 3.1 Simulation results of the main process (scenario 1 & 2)

This subsection presents the key results for the main process, starting with some general simulation outcomes. A detailed overview of the main input values and flow characteristics for the Main Process (Scenario 1) is provided in Appendix Table A1.

Three major outlet streams leave the process, namely S-156 containing depleted medium, S-154 representing the waste stream, and DS-102 which contains packaged biomass product (see Figure 4). S-156 only contained “Depleted Medium” at 15.0 °C and 1.013 bar, while S-154 contained “Buffer Solution,” “Depleted Medium,” and “Impurities” at 10.8 °C and 1.013 bar. The discrete stream DS-102 consisted of 2,091.69 entities with a total mass stream of 2,112.61 kg/batch. Each entity comprised 1 kg of material from the inlet stream S-155, containing the biomass product, and 10 g of the “Container” component, making up the packaging and introduced via DS-101. For the 50:50 split variant, the filling operation in P-23 / FL-101 finished 734.67 h after the batch started, and the latest operation, purging of storage tank P-20 / V-103, finished 736.92 h after the start, marking the end of the process. For the 90:10 split variant, the filling operation in P-23 / FL-101 finished 734.01 h after the batch had started, and the purging of storage tank P-20 / V-103 finished 736.26 h after the start. In the following, the three process stages, media preparation, cell expansion, and downstream processing, as well as the CIP and heating/cooling duties, are investigated.

#### 3.1.1 Media preparation

First, the results for *Scenario 1: Main Process, 90:10 split* are shown. Sizing calculations for the larger tank V-101 before sterile filtration yielded a volume of 20,000.00 L, accounting for the 90% maximum working volume. The smaller tank V-102, before heat sterilization, was determined to have a volume of 2,222.22 L. The filtration units DE-101 and DE-102 required a filter area of 20 m^2^ each, equivalent to two cartridges of 10 m^2^. In case of the heat sterilization in P-3 / ST-101, a cooling duty of 0.48 kW requiring a coolant flow of 82.0 kg/h and a heating duty of 5.0 kW requiring a heating agent flow of 8.54 kg/h were determined. The temperature of S-102 after mixing in MX-101 was 26.0 °C. The following cooling operation in V-110 required a cooling duty of 80.79 kW while taking 220.26 min at a cooling rate of 0.1 °C/min. The “React” operation finished 9.17 h after the start of the batch, marking the beginning of the cell expansion in P-8 / SFR-101.

For *Scenario 2: Main Process, 50:50 split*, the storage tanks V-101 and V-102 had to hold 10,000.00 L of medium broth each, resulting in a suggested vessel volume of 11,111.11 L based on 90% maximum working volume. For the sterile filtration in DE-101 and DE-101, a filter area of 10 m^2^ each was determined – the size of one cartridge. For heat sterilization in P-3 / ST-101, a cooling duty of 2.41 kW requiring 415.6 kg/h of the cooling agent and a heating duty of 25.4 kW requiring 43.31 kg/h of the heating agent were determined. The stream temperature of S-102 after mixing in MX-101 was 30.0 °C. The cooling operation in V-110 required 30.86 kW while taking 260.12 min at a cooling rate of 0.1 °C/min. The “React” operation in V-110 was finished 9.84 h after the start of the batch.

#### 3.1.2 Cell expansion

The cell expansion stage took just over 30 days or 724.8 h. Apart from the purge operations beforehand and the CIP operations afterwards, the cell expansion took place between the start of the “Transfer In” operation in SFR-101 and the end of the “Transfer Out” operation in BR-102. The respective times were 9.17 h to 734.01 h for the 90:10 split and 9.84 h to 734.67 h for the 50:50 split in reference to the start of the batch. The purging operations, together with the air supply during fermentation, resulted in the use of 11,506.78 kg of “Synthetic Air” in the cell expansion stage, according to the *Materials & Streams Report*. In contrast, the amount of “Air” used for the entire process due to purging and filling/emptying of vessels was just over 33.3 kg according to the same report. The coolant flow necessary to keep the temperature steady at 37 °C inside the six vessels as a result of heating due to the dissipation of agitation energy is listed in Table 7 together with the respective total power demand, taken from the “Ferment” operations and based on the provided fallback values of the specific power demands. The corresponding cooling requirements are directly linked to the agitation power input and the resulting power density (*P/V*), which become increasingly important at larger bioreactor volumes. Since the values in Table 7 represent operational loads during reactor operation rather than time-averaged batch consumption, they can also be interpreted as the peak utility demands associated with each individual vessel. The composition of S-145, leaving BR-102 for downstream processing, is shown in Table 4 and contains “Biomass,” “Depleted Medium,” and “Impurities.”

**Table 7:** Instantaneous coolant flow rates and total power demand during active operation for each of the six reactors in the cell expansion stage.

Vessel	Specific power in kW/m^3^	Coolant flow rate in kg/h	Total power in kW
SFR-101	3.00	0.04	0.0003
SFR-102	3.00	0.69	0.0047
RBS-101	3.00	10.78	0.073
RBS-102	3.00	108.13	0.73
BR-101	0.50	77.12	0.97
BR-102	0.50	748.58	9.69

The values depend on the specific power demand, which was set to the provided fallback value for each vessel type.

#### 3.1.3 Downstream processing

The DSP part took 4.75 h. The initial cooling to 25.0 °C in P-18 / HX-101 led to a coolant flow rate of 8,892.12 kg/h. The power demand of the centrifuge P-17 / DS-101 was determined to be 35.64 kW, and with a power to heat dissipation of 25% (standard value from the software), the coolant flow rate necessary to achieve 15.0 °C outlet temperature was 8936.11 kg/h. Both streams leaving the centrifuge, waste stream S-147 and product-containing stream S-151, contained “Biomass,” “Depleted Medium,” and “Impurities,” albeit in different proportions. Although the majority of “Depleted Medium” and “Impurities” partitioned into waste stream S-147, “Depleted Medium” still constituted the largest mass percentage in the product-containing stream S-151 (60.60%). The majority of “Biomass” was part of S-151 (constituting 38.39% of the stream), while only a fraction was contained in S-147. The latter was guided through the filtration unit procedure P-19 / DE-103 with a filter area of 20 m^2^ or two cartridges. The filtration unit removed “Biomass” and “Impurities” completely, while the amount of “Depleted Medium” remained the same. The required size of the receiving tank V-103 was determined to be 14,402.33 L, with a maximum working volume of 90%. S-151 was cooled down to 10.8 °C during washing in P-21 / WSH-101, becoming S-153. S-153 had the same composition as S-155, as mixing with different additives in the extrusion operation was not modeled. The “Extrusion” unit procedure P-22 / XD-101 served only as a placeholder for a downstream processing step likely to be part of the CM production. The coolant flow rate in P-22 / XD-101 was 0.31 kg/h to achieve a final stream temperature of 4.0 °C. The discrete stream DS-101 “Packaging” was determined to contain 2,091.69 entities of 10 g of “Container” or a total amount of 20.92 kg/batch.

#### 3.1.4 CIP procedures and heating / cooling duties

The amounts of washing solutions used for CIP operations are listed in Table 8. Due to differences in equipment sizes in the media preparation stage, the 50:50 split required 3.14% more Acidic Wash Solution (AWS) and Sanitizing Wash Solution (SWS), and 5.75% more Caustic Wash Solution (CWS) compared to the 90:10 split. An increase was also observed in the use of “Water,” which was utilized for CIP operations. According to the *Materials & Streams Reports*, the 50:50 split variant required 60,514.80 kg/batch, while the 90:10 split variant required 58,778.74 kg/batch. This increase of 2.95% can be attributed entirely to the CIP duties. The *Energy Reports* display the power demand and cooling/heating agent usage of the process. For the cooling operation in P-5 / V-110, the total power demand was 113.36 kW∙h for the 90:10 split and 133.79 kW∙h, for the 50:50 split. Similarly, the demand for heating agents and coolants was much higher for the 50:50 split during media preparation, as shown in the data presented in Table 8 showing a five-fold demand compared to 90:10. However, most coolant was used in the form of “Chilled Water” during cell expansion and DSP stage for both split variants.

**Table 8:** Comparison of CIP and heating/cooling utility demands across the three process scenarios.

Agent / mixture	Amount in kg/batch for Scenario 1: main process, 90:10 split	Amount in kg/batch for Scenario 2: main process, 50:50 split	Amount in kg/batch for Scenario 3: local-scale variation
AWS	2,001.07	2,063.92	826.94
CWS	3,842.06	4,063.10	1,003.20
SWS	1,000.01	1,031.42	413.25
Water	58,778.74	60,514.80	15,236.23
Cooling Water	410.23	2,078.01	41.02
Chilled Water	163,111.91	163,111.70	30,750.46
Steam	42.72	216.53	4.27

The values are derived from the *Materials & Streams Report*s. Differences result from different equipment sizes in the media preparation stage between Scenario 1 and Scenario 2, and from the reduced overall process scale in Scenario 3.

### 3.2 Simulation results of process variations

This subsection presents the results from the process variations *Scenario 3: Local-Scale Variation* and *Scenario 4: Cell Expansion Variation*, described in section 2.2. The subdivision of the main process into standalone processes is not considered separately.

#### 3.2.1 Scenario 3: local-scale variation

The three major outlet streams are S-156 containing depleted medium, S-154 representing the waste stream, and S-143 which contains cooled biomass. The flow rates decreased tenfold, and the compositions were the same as for *Scenario 1: Main Process, 90:10 split* (except for small variations in the last digits). This was found for all streams, as expected. The “Cool” operation in EC-101 was finished 614 h after the batch’s start, and the “Purge” operation in V-103 after 616.50 h, marking the end times. For the cell expansion stage, the numbers for SFR-101 through BR-101 were essentially identical to *Scenario 1: Main Process, 90:10 split*, as expected. As can be checked with the *Materials & Streams Report* and the *Energy Report* included in the Supplementary Materials, many numbers presented in Section 3.1 for *Scenario 1: Main Process, 90:10 split* have just decreased tenfold, as was to be expected after removing the final cell expansion stage P-14 / BR-102, while others did not scale proportionally. In particular, CIP and cooling/heating related duties and material demands did not scale proportionally. This can be seen in Table 8, which compares the utility demands across all three Scenarios discussed so far. The filtrations DE-101, DE-102, and DE-103 each used a single cartridge of 10 m^2^ filter area. The tank sizes were determined as follows: 2,000.00 L for V-101, 222.22 L for V-103, 2,223.03 L for V-110, and 1,440.23 L for V-103.

#### 3.2.2 Scenario 4: cell expansion variation

Data from the cell expansion variation file was compared with data from the standalone cell expansion file from *Scenario 1: Main Process, 90:10 split*. With five bioreactors instead of six, the fermentation time was reduced by 5 days from 720 h to 600 h for the cell expansion variation. The composition of the outlet stream S-107 for the standalone process was essentially identical to S-121, as desired. In Table 9, the cell expansion variation was compared with the standalone process in terms of supplied agents/mixtures for CIP, heating/cooling, and aeration. Agents for CIP showed an increase of 24.7% while the other duties benefited from the reduced number of reactors in cell expansion variation, which was also supported by a lower power demand of 1,615.61 kW∙h/batch compared to 1,720.08 kW∙h/batch for the standalone process.

**Table 9:** Comparison of CIP, heating/cooling, and aeration material supply for cell expansion variation and standalone process.

Agent / mixture	Amount in kg/batch for standalone	Amount in kg/batch for variation	Difference in % (basis: standalone)
AWS	692.33	863.44	24.7
CWS	688.05	858.09	24.7
SWS	345.98	431.49	24.7
Synthetic Air	11,574.41	10,387.53	10.3
Water	10,358.13	12,918.09	24.7
Chilled Water	244,520.49	230,880.63	5.6

The values are derived from the *Materials & Stream*s and *Energy Reports*.

## 4 Discussion

This paper establishes a process-wide model for the production animal cell biomass that integrates media preparation, sequential cell expansion, downstream processing, and auxiliary operations into one coherent flowsheet. The approach was chosen to generate complete mass and energy balances under clearly defined assumptions and to create a transparent platform design that enables comparison of different production strategies but can also serve as a reference for future work. Four production scenarios were simulated and compared.

*Scenario 1: Main Process, 90:10 split,* and *Scenario 2: Main Process, 50:50 split*, only differ in the volumetric split between filtration and heat sterilization during media preparation. Simulation results show an increase in power demand associated with heat sterilization, as well as heating and cooling agents required for the 50:50 split. Additionally, the amount of washing solutions needed for CIP was increased due to differences in equipment sizes for the media preparation stage. This suggests that filtration is the preferred sterilization method for media preparation. However, these increased energy demands need to be weighed against the increased filter requirements for the 90:10 split. A future techno economic assessment and life cycle assessment based on the presented simulation can be linked here to provide accurate information on economic feasibility and environmental impact. The assessment of the preferred split between sterilization methods will most likely depend on heating costs, CIP material costs, initial equipment costs, and sterile filtration consumable costs, from an economic perspective. From a sustainability perspective, it will most likely depend on the environmental impact of filtration cartridges versus the higher energy and water demand of heat sterilization, along with the respective waste treatment for both methods.

Simulation of *Scenario 3: Local-Scale* Var*iation* revealed a disproportional reduction of many process utility materials, such as coolant and washing solutions. While the product amount was reduced by a factor of 10 compared to *Scenario 1: Main Process, 90:10 split*, the amounts of water, chilled water, AWS, CWS, and SWS were only reduced by 59–83%. The production time was only reduced by 5 days, from about 31 days to about 26 days. While lower capital costs can be expected from a smaller facility, the simulations indicate that consumable demand and production time favor larger production bioreactors.

From the simulation of *Scenario 4: Cell Expansion Variation*, it is notable that production facilities utilizing high cell density cryopreservation, which provides a higher volume first-stage inoculum, gain a time advantage (in this particular scenario, 5 days). However, cell banking is not considered in the model. Preparing the same number of 200 mL cryo-bags requires more resources in pre-production culturing than for 2 mL cryo-vials. Furthermore, the technology for direct revival and viability maintenance in large bioreactors is still highly immature, so it cannot yet be directly applied to industrial CM production, but it can be considered as a potential long-term option for CM intensification if future developments prove successful. Elimination of the single-use equipment in the cell expansion stage led to about 25% increase in the water, AWS, CWS, and SWS used for CIP compared to *Scenario 1: Main Process, 90:10 split*. While both cost and sustainability advantages of the single-use equipment were reported for biopharmaceuticals (29), more CM-specific data are required for a final assessment. Many advantages of single-use equipment are linked with the specifics of the respective processes, where flexible processes are often desired for biopharmaceuticals. This enables one facility to produce multiple products with comparably low output per product. For CM production, this flexibility might not be needed because a reliable and robust process with high production output is desired. Therefore, STRs might still be preferred despite higher consumption of the CIP-related utilities, as they can be automated and do not need to be set up manually for each run.

The results support that media composition and preparation remain critical factors in process performance. Despite their low concentration in the synthetic medium, the high cost of proteins and growth factors dominates resource demand, as was shown in earlier cost and sustainability analyses (3). The sterilization strategy introduces an additional layer of complexity. While steam-based sterilization is straightforward to model, it imposes high energy and water loads, whereas sterile filtration reduces utilities but requires significant membrane area. Both routes highlight the trade-offs between operational feasibility and resource efficiency. The cell expansion cascade was parameterized with conservative mammalian culture kinetics, resulting in a maximum density of 5 × 10^7^ cells/mL in the 20,000 L STR. Higher densities or shorter doubling times would directly increase the overall process productivity, but such improvements require targeted advances in cell line engineering and process control. For downstream processing, a simplified centrifugation-washing-extrusion sequence was modeled to capture the essential mass flows. While this abstraction is sufficient for quantifying biomass recovery, it does not address product quality parameters such as texture, lipid integration, or sensory properties. These aspects will need to be incorporated into future iterations once reproducible data becomes available.

An essential contribution of the model is the explicit quantification of waste and utility streams. Depleted medium, impurities, CIP solutions, and cooling demand represent significant outputs that define both the environmental footprint and opportunities for valorization.

At the 10,000 L scale, heat generation associated with agitation becomes a significant contributor to the overall cooling demand. Mechanical energy introduced by the impeller is ultimately dissipated as heat within the culture medium, making the agitation power input (*P*) and corresponding power density (*P/V*) important scale-up parameters. Although large-scale bioprocesses often reduce *P/V* relative to laboratory systems to limit shear stress and energy consumption, sufficient mixing intensity must still be maintained to ensure homogeneous nutrient distribution and oxygen transfer (3). Consequently, even moderate *P/V* values at 10,000 L can result in substantial absolute heat loads due to the large reactor volume. In addition, thermal management becomes increasingly challenging during scale-up because heat generation scales approximately with reactor volume, whereas heat removal capacity scales primarily with heat-transfer surface area. As reactor dimensions increase, the surface-area-to-volume ratio decreases, reducing the relative efficiency of cooling. Therefore, large-scale CM bioreactors may require dedicated cooling strategies despite the relatively low operating temperature of mammalian cell culture (~37 °C). This creates an important trade-off between oxygen transfer performance, agitation intensity, shear protection, and cooling requirements, emphasizing the need for integrated hydrodynamic and thermal design during scale-up.

Although recycling strategies were not implemented here, the model provides the necessary basis for integrating circularity measures such as media regeneration, CO₂ reuse, or anaerobic digestion of biomass side streams (6). This is crucial because both the cost and demand of the medium are high, making medium recycling likely necessary for achieving economically viable CM production at industrial scale (30). Incorporating media recycling would primarily affect the media preparation stage, as this step is directly responsible for media formulation and sterilization. Previous research for example suggested that medium recycling could significantly reduce the use of sterile purified water which represents an energy-intensive resource (31). Consequently, the material demand and, in part, the utility demand would be reduced depending on the employed strategy (32) and the recycled fraction. Recycling rates between 50 and 90% were typically explored in the CM literature, although usually only at laboratory scale and with limited multi-cycle reuse data (33, 34). Here, 50% recycling rate does not automatically translate into 50% reduction of all media preparation inputs. While raw material demands for medium components would reduce roughly proportionally, utility demands are expected to scale disproportionally, due to the additional process steps required for separation, filtration, conditioning, and quality control of the recycled medium. These steps introduce additional energy consumption, process complexity, and potential losses depending on the recycling strategy employed. As different strategies vary greatly regarding their energy, infrastructure, and consumables demands (35), and none of them has yet been applied to mammalian cell cultures (31) or has been developed into a commercialized, ready-to-use system (36), each should be considered separately to evaluate its impact on the overall process.

The limitations of the present model are mainly related to data availability. The synthetic medium does not capture the diversity of proprietary formulations; cell growth was modeled with fixed stoichiometries and deterministic kinetics; and downstream operations were simplified to generic steps. Although the key process parameters, such as growth kinetics, achievable cell densities, and media use, were based on the available mammalian cell culture literature, they were not experimentally validated at pilot or industrial scale. A further limitation relates to the biological feasibility of large scale suspension cultivation for cultured meat applications. Many relevant animal cell types are naturally anchorage dependent and require adaptation to suspension systems, potentially affecting phenotype, differentiation behavior, and productivity (1, 5). Although suspension adapted mammalian cell systems are established in biopharmaceutical manufacturing, their transferability to food relevant animal cells remains insufficiently validated at industrial scale (3). Furthermore, publicly available large-scale data for cultivated biomass production are very limited, particularly regarding the long-term culture stability and scale-dependent effects, meaning that the biological and technical feasibility of literature-derived assumptions from the laboratory-scale cell culture is yet to be determined. However, these assumptions were necessary to construct a transparent baseline, but they introduce uncertainty in extrapolating to commercial scales. Consequently, the results should be interpreted primarily as a comparative and exploratory framework rather than a precise prediction of commercial process performance.

## Data Availability

The original contributions presented in the study are included in the article/ Supplementary material, further inquiries can be directed to the corresponding author.
