# Supplementary material for: Decoding cultured meat manufacturing: a full process model to identify scale-up bottlenecks
Source: Front Nutr. 2026 Jun 29;13:1844185. doi: 10.3389/fnut.2026.1844185 (PMC13357269; doi:10.3389/fnut.2026.1844185)
Supplement: Supplementary file 1 [file Table_1.DOCX]

Supplementary Material

Table A.1: Overview of main input values and flow characteristics used in the process model of Scenario 1: Main Process, 90:10 split.

| **Parameter** | **Value** | **Unit** | **Comment** |
| --- | --- | --- | --- |
| Recipe Batch Time | 736.51 | h | As defined in SuperPro Designer |
| Number of Batches per Year | 10 | batches/year | Based on total yearly runtime |
| Annual Operating Time | 7,360.59 | h/year | Total utilized time for production |
| Total Liquid Volume (early streams) | 18,900 – 19,000 | L/batch | Includes major early streams like S-101 to S-149 |
| Volumetric Flow Rate (approx. P-15) | ~2,600 | L/h | Estimated from volume/time |
| Total Biomass Input | 1,990.76 | kg/batch | Biomass entering DSP from upstream |
| Final Product Biomass | 1,891.22 | kg/batch | Final extracted biomass |
| Solid Content (approx.) | ~10.5% | kg/kg or % | Biomass as a share of total input for DSP |
| Depleted Medium | 15,926.07 | kg/batch | Total liquid removed post-culture |
| Buffer Solution | 2,004.72 | kg/batch | Used for washing & purification |
| Impurities (input) | 995.38 | kg/batch | Non-cellular waste content input |
| Impurities (waste stream) | 945.61 | kg/batch | Portion ending up in waste |
| Water Consumption | 58,778.74 | kg/batch | Total water used in the process |
| Estimated Cell Number | 1e12 – 1e13 | cells/batch | Rough estimate, depending on cell size |
| Temperature Range | 4 – 37 | °C | Cooling and heating stages |
| Pressure Range | 1.01 – 2.01 | bar | Mild overpressure conditions |
| Containers (kg) | 20.92 | kg/batch | Packaging material in kg |
| Containers (units) | 2,091.69 | units/batch | Based on output units |
| Final Product Stream Volume | 2,022.03 | L/batch | Final output stream volume (S-102) |
| Total Batch Mass (e.g., S-101) | 18,912.21 | kg/batch | Combined mass of the major input stream |
| Total Batch Volume (e.g., S-101) | 18,982.64 | L/batch | Combined volume of the same stream |
| CWS (Caustic Wash Solution) | 3,842 | kg/batch | Distributed across unit operations |
| SWS (Sanitizing Wash Solution) | 1,000 | kg/batch | Sanitized for reuse |
| AWS (Acidic Wash Solution) | 2,001 | kg/batch | Auxiliary water for processes |
| Total Component Mass In | 35,490.63 | kg | From the overall material balance |
| Total Component Mass Out | 34,445.09 | kg | From the overall material balance |
| Overall Waste Stream Volume | 12,962.09 | L | Main liquid waste from P-20 |
| Bioreactor Size | 25,000 | L | Nominal reactor volume |
| Working Volume Assumption for STRs | 80% | % | Typical SuperPro design assumption |
| Effective Working Volume | 20,000 | L | 25,000 L × 0.8 |
| Processed Liquid per Batch (total) | 18,900 – 19,000 | L/batch | Total liquid volume processed in DSP |
| Solids Content in Stream (S-101) | ~10.5% | kg/kg | Biomass share in total input mass |
